# Tumor necrosis factor receptor regulation of peripheral node addressin biosynthetic components in tumor endothelial cells

**DOI:** 10.3389/fimmu.2022.1009306

**Published:** 2022-09-15

**Authors:** Anthony B. Rodriguez, Geoffrey Parriott, Victor H. Engelhard

**Affiliations:** Carter Immunology Center and Department of Microbiology, Immunology, and Cancer Biology, University of Virginia School of Medicine, Charlottesville, VA, United States

**Keywords:** blood endothelial cells, high endothelial venules, PNAd, B16, carbohydrate sulfotransferase, checkpoint immunotherapy, tertiary lymphoid structure

## Abstract

Tumor-associated tertiary lymphoid structures are ectopic lymphoid aggregates that have considerable morphological, cellular, and molecular similarity to secondary lymphoid organs, particularly lymph nodes. Tumor vessels expressing peripheral node addressin (PNAd) are hallmark features of these structures. Previous work from our laboratory demonstrated that PNAd is displayed on intratumoral vasculature of murine tumors, and its expression is controlled by the engagement of lymphotoxin-α_3_, secreted by effector CD8 T cells, with tumor necrosis factor receptors (TNFR) on tumor endothelial cells (TEC). The goals of the present work were: 1) to identify differences in expression of genes encoding the scaffolding proteins and glycosyl transferases associated with PNAd biosynthesis in TEC and lymph node blood endothelial cells (LN BEC); and 2) to determine which of these PNAd associated components are regulated by TNFR signaling. We found that the same genes encoding scaffolding proteins and glycosyl transferases were upregulated in PNAd^+^ LN BEC and PNAd^+^ TEC relative to their PNAd^neg^ counterparts. The lower level of PNAd expression on TEC vs LN BEC was associated with relatively lower expression of these genes, particularly the carbohydrate sulfotransferase *Chst4*. Loss of PNAd on TEC in the absence of TNFR signaling was associated with lack of upregulation of these same genes. A small subset of PNAd^+^ TEC remaining in the absence of TNFR signaling showed normal upregulation of a subset of these genes, but reduced upregulation of genes encoding the scaffolding proteins podocalyxin and nepmucin, and carbohydrate sulfotransferase Chst2. Lastly, we found that checkpoint immunotherapy augmented both the fraction of TEC expressing PNAd and their surface level of this ligand. This work points to strong similarities in the regulation of PNAd expression on TEC by TNFR signaling and on LN BEC by lymphotoxin-β receptor signaling, and provides a platform for the development of novel strategies that manipulate PNAd expression on tumor vasculature as an element of cancer immunotherapy.

## Introduction

Trafficking of leukocytes, including T- and B-cells, into lymphoid and inflamed non-lymphoid tissues involves sequential interactions of a set of homing receptors (HR) on leukocytes with cognate ligands on blood endothelial cells (BEC) (1). During an immune response, effector T cells acquire the ability to enter inflamed tissues by upregulating HR that bind to ligands that are upregulated on activated BEC. HR and HR ligands that are utilized for T-cell infiltration into tumors have been identified (2–7). However, tumor endothelial cells (TEC) express HR ligands at low levels (8–10), and a number of studies have showed a correlation between the levels of HR ligands on the tumor vasculature and the numbers of intratumoral T cells (9, 11–16). A better understanding of how HR ligands are regulated in TEC may provide significant opportunities to increase the number of intratumoral T cells.

Since naïve T cells do not generally enter peripheral tissue, it had been assumed that all intratumoral lymphocytes are effectors that differentiate in tumor-draining LN and home to the tumor thereafter. However, naïve T cells infiltrated tumors that had been genetically modified to secrete homotrimeric lymphotoxin-α (LTα_3_) (17, 18) or LIGHT (19). Similar results were also obtained through the intratumoral injection of either homeostatic chemokine CCL21 (20, 21) or DCs genetically modified to express this molecule (22). Naïve T cells enter lymph nodes (LN) based on their expression of L-selectin and CCR7, which bind to peripheral node addressin (PNAd) and the chemokines CCL19/CCL21, respectively (23). These HR ligands are normally expressed on specialized LN blood vessels called high endothelial venules. However, they have been detected in a variety of human tumors, and their presence is associated with a positive clinical prognosis (24–30). Recently, our laboratory demonstrated that PNAd and CCL21 were co-expressed on a small fraction of blood vessels in murine melanomas and lung carcinomas growing in multiple anatomical locations (31). The primary source of CCL21 was cancer associated fibroblasts, and to a lesser extent, TEC. PNAd^+^ CCL21^+^ vessels developed spontaneously, and supported the infiltration of naive CD8 T cells that differentiated into functional effectors after intratumoral activation and significantly delayed tumor outgrowth (31, 32). Thus, PNAd^+^ CCL21^+^ intratumoral vessels contribute to anti-tumor immunity by generating a self-sustaining infiltration of naïve T cells into the tumor mass.

**Figure 1:**
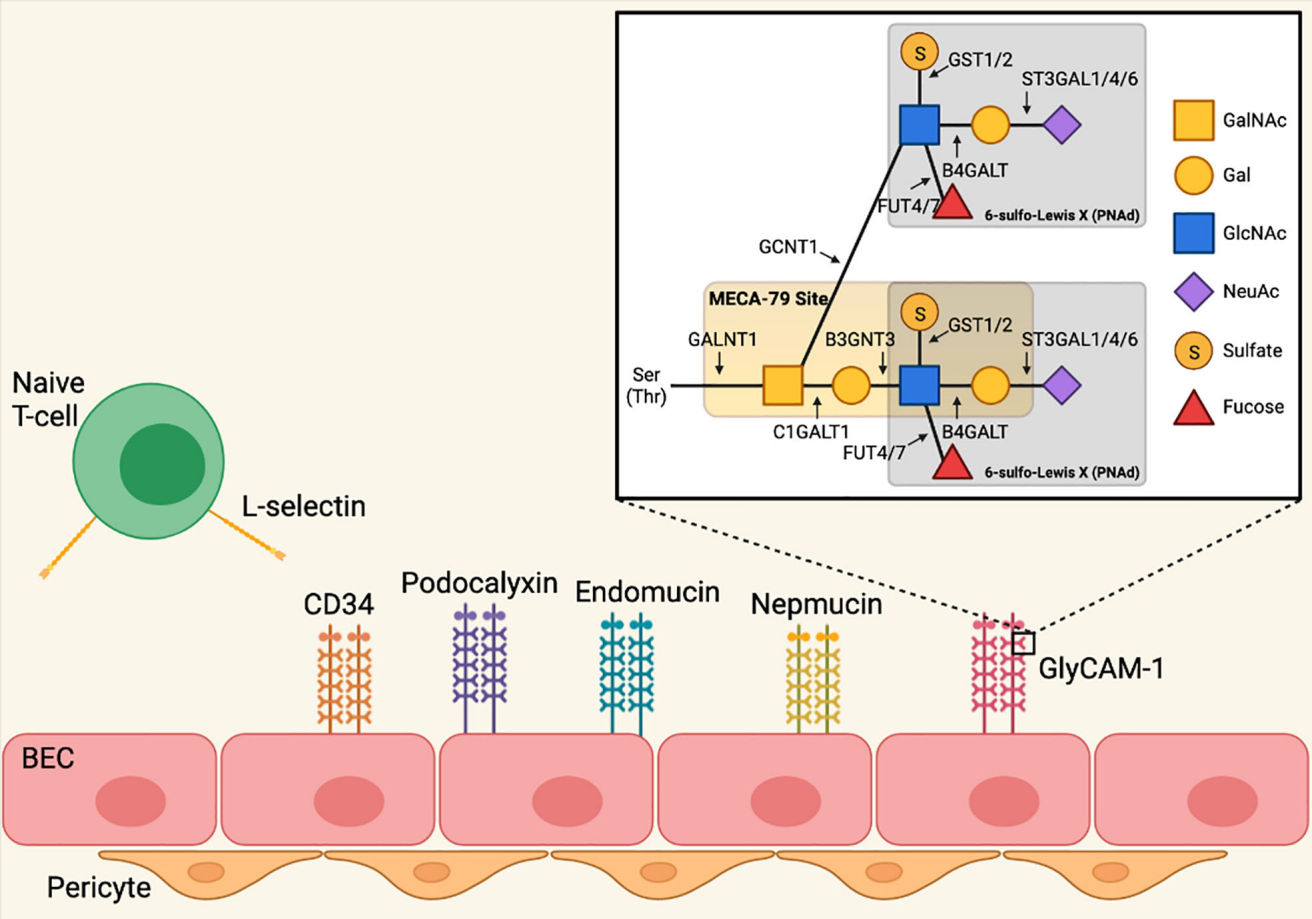
Biosynthesis of peripheral node addressin in lymph node blood endothelial cells. PNAd refers to the 6-sulfo sialyl Lewis X carbohydrate structure that is displayed on *O*-linked glycans that decorate multiple mucin-domain containing scaffolding proteins. Generation of 6-sulfo sialyl Lewis X involves a series of post-translational modifications mediated by glycosyl transferases. Polypeptide *N*-acetylgalactosaminyltransferase 1 (GALNT1) initiates formation of the *O*-linked glycan by attaching a *N*-acetyl-galactosamine (GalNAc) residue to serine and/or threonine (35). *O*-glycans are biantennary structures consisting of Core 1 and Core 2 branches. Glycoprotein-*N*-acetylgalactosamine 3-beta-galactosyltransferase 1 (C1GALT1, Core 1 synthase) creates the core 1 branch by attaching a galactose (Gal) residue to the O-linked GalNAc residue (36, 37), and this branch is extended by the addition of *N*-acetylglucosamine (GlcNAc) by beta-1,3-*N*-acetylglucosaminyltransferase 3 (B3GNT3) (37). The Core 2 branch is created by Beta-1,3-galactosyl-O-glycosyl-glycoprotein beta-1,6-*N*-Acetylglucosaminyltransferase 1 (GCNT1, Core 2 synthase), which attaches a GlcNAc residue to the *O*-linked GalNAc (38). The GlcNAc residues of the Core 1 and Core 2 branches are each modified in 3 additional ways. Galactose/N-acetylglucosamine/*N*-acetylglucosamine 6-O-sulfotransferase 1 (GLCNAC6ST-1) and 2 (GLCNAC6ST-2) attach sulfate onto the Core 1 and Core 2 GlcNAc residues, although GLCNAC6ST-1 acts only inefficiently to modify Core 1 (39–42). Alpha- (1, 3)-fucosyltransferase 4 (FUT4) (43) and 7 (FUT7) (44) attach fucose to both Core 1 and Core 2 GlcNAc residues. Finally, beta-1,4-galactosyltransferase (B4GALT) attaches Gal residues to GlcNAc in both branches (45), which are then further modified by CMP-N-acetylneuraminate-beta-galactosamide-alpha-2,3-sialyltransferase 1 (ST3GAL1) (46), 4 (ST3GAL4) (47), and 6 (ST3GAL6) (48), which attach *N*-acetylneuraminic acid (NeuAc) to Gal. This completes formation of the 6-sulfo sialyl Lewis X structure that is recognized by L-selectin expressed on naïve and central memory T- and B-cells. Image created with BioRender.com.

PNAd refers to the 6-sulfo sialyl Lewis X carbohydrate structure that is displayed on *O*-linked glycans that decorate multiple mucin-domain containing scaffolding proteins, including GlyCAM-1, CD34, sgp200, podocalyxin, endomucin, and nepmucin, and recognized by L-selectin (33, 34). Generation of 6-sulfo sialyl Lewis X involves a series of post-translational modifications, described in detail in **Figure 1**. The glycosyl transferases GALNT1, C1GALT1, B3gnt3, and GCNT1 construct a biantennary structure (35–38). The terminal GlcNAc residues of the Core 1 and Core 2 branches are each modified in 3 additional ways: fucosylation by FUT7 and FUT4 (43, 44); galactosylation and sialylation by B4GALT and one of several sialyl transferases (45–48); and sulfation by GlcNAc6ST-1 and GlcNAc6ST-2. While GLCNAC6ST-2 efficiently attaches sulfate onto both Core 1 and Core 2 GlcNAc residues, GLCNAC6ST-1 acts primarily on Core 2, and only inefficiently modifies Core 1 (39–42). This completes formation of the 6-sulfo sialyl Lewis X structure that is recognized by L-selectin expressed on naïve and central memory T- and B-cells.

In adult LN, transcripts of genes encoding the scaffolding protein GlyCAM-1 *(Glycam1)*, and the transferases B3GNT3 (*B3gnt3)*, GCNT1 (*Gcnt1)*, GLCNAC6ST-1 (*Chst2)*, GLCNAC6ST-2 (*Chst4)*, and FUT7 (*Fut7)* are elevated in PNAd^+^ compared to PNAd^neg^ BEC, while transcripts of genes encoding the scaffolding proteins CD34 (*Cd34)*, podocalyxin (*Podxl)*, endomucin (*Emcn*), and nepmucin *(Cd300lg)* are comparable (49, 50). Expression of *Glycam1*, *Gcnt1*, *Chst2*, *Chst4*, and *Fut7* is also dependent on continuous engagement of the lymphotoxin-β receptor (LTβR) expressed on LN BEC with lymphotoxin-α_1_β_2_ (LTα_1_β_2_) expressed on DCs (50–52), while *B3gnt3* is regulated independently of LTβR signaling through an unknown pathway (50). *Chst2* (53) and *Chst4* (54) can also be induced in cultured monocytes and endothelial cells, respectively, by TNFα, leading to expression of PNAd, but the impact of TNFα on expression of other PNAd biosynthetic components is unknown.

Previous work from this lab demonstrated that PNAd expression on murine melanoma TEC was not induced by either DC or LTβR signaling, and was instead controlled by effector CD8 T cells secreting LTα^3^, which signaled through TNF receptors (TNFR) expressed on TEC (31). Here, we demonstrate that TEC expressed key PNAd glycosyl transferases and scaffolding proteins normally found in LN BEC, albeit at lower levels. We also determined the mechanisms by which TNFR regulated their expression, and that checkpoint immunotherapy augments PNAd expression on the tumor vasculature. These findings provide significant insight into the basis for PNAd biosynthesis in TEC.

## Results

### PNAd is expressed at substantially lower levels on tumor endothelial cells than on lymph node blood endothelial cells

**Figure 2:**
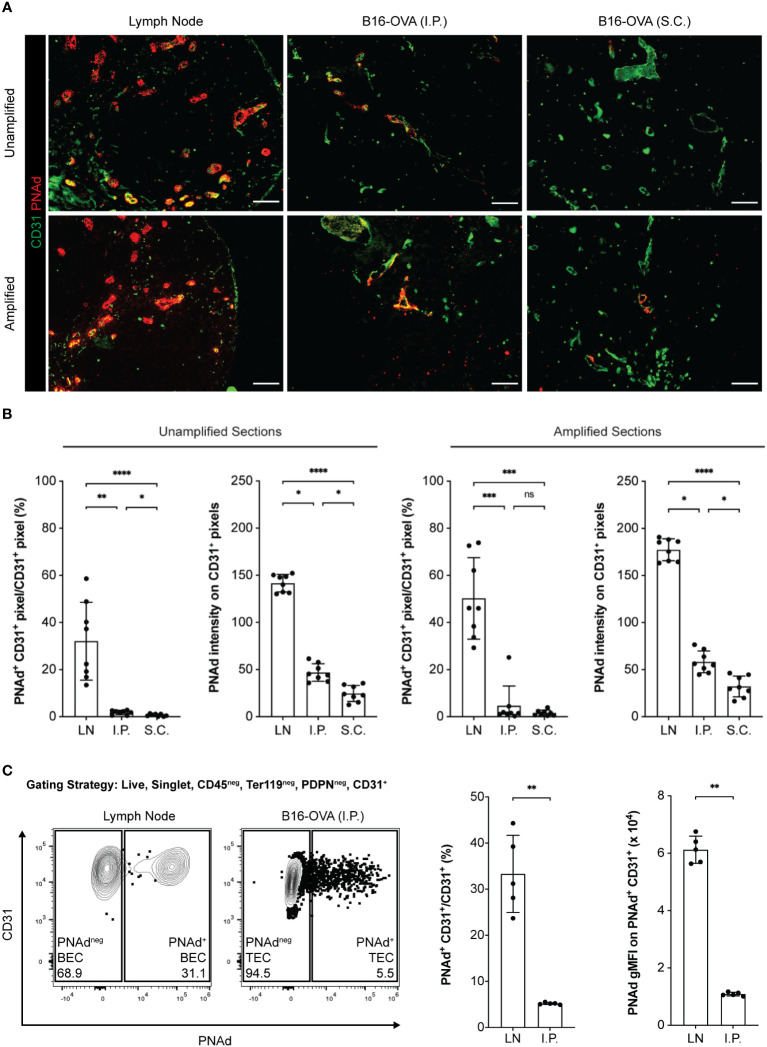
PNAd expression is significantly lower on tumor-associated endothelium than on lymph node endothelium. C57BL/6 mice were S.C. or I.P. injected with B16-OVA cells. Tumors were harvested 14 days later. LN were harvested from non-tumor-bearing mice. Resected tissues were prepared for IF microscopy **(A, B)** or flow cytometry **(C)** as described in Methods. **(A)** Representative images of unamplified and tyramide signal amplified LN and tumor sections stained with indicated markers. Scale bar = 100 µm. **(B)** Quantitative image summary data for unamplified and amplified tissue sections. PNAd percentages and pixel intensities were calculated on a CD31^+^ mask. Data represents two experiments, n = 8 sections per group. **(C)** Representative flow cytometry plot, percentages, and gMFI intensities of PNAd on CD45^neg^ Ter119^neg^ PDPN^neg^ CD31^+^ endothelial cells in CD45^+^ depleted LN and I.P. tumors suspensions. PNAd gMFIs were calculated on cells gated above the fluorescence minus one (FMO) control. Data represents one experiment, n = 5 LN or tumors per group. **(B, C)** Results are mean standard deviation (SD) analyzed by unpaired Welch’s t-test. ns: p>0.05, *p<0.05, **p<0.01, ***p<0.001, and ****p<0.0001.

Transplantable murine tumors growing intraperitoneally (I.P.) and subcutaneously (S.C.) develop CD31^+^ blood vessels that express PNAd (31). By immunofluorescence microscopy (IF), ~30% of CD31^+^ BEC pixels in LN sections co-stained for PNAd, while ~1.8% and ~0.7% of CD31^+^ TEC pixels in I.P. and S.C. tumor sections did so, respectively (**Figure 2A** **)**. The staining intensity of PNAd^+^ pixels on TEC was also significantly lower than on LN BEC. However, the fraction of CD31^+^ pixels that were PNAd^+^ was significantly higher in I.P. than S.C. tumors, and their staining intensity was also significantly higher. Tyramide signal amplification enables detection of low-abundance targets by IF. In tyramide amplified sections, ~50% of CD31^+^ BEC pixels in LN co-stained for PNAd, while ~4% and ~1.6% of CD31^+^ TEC pixels in I.P. and S.C. tumors, respectively, did so (**Figure 2A** **)**. PNAd staining intensity in tyramide amplified tumor sections was still significantly lower than in amplified LN sections, and still significantly higher in amplified I.P. sections than in amplified S.C. sections. By flow cytometry, which has the capacity to detect low-abundance targets on cells without amplification, PNAd was expressed at a uniformly high levels on ~30% of CD31^+^ LN BEC. In contrast, ~5% of I.P. CD31^+^ TEC stained for PNAd, and the geometric mean fluorescence intensity (gMFI) of PNAd on these cells was significantly lower and more variable relative to PNAd^+^ LN BEC (**Figure 2**). Overall, these results demonstrate that TEC express PNAd less frequently and at substantially lower levels relative to LN BEC, although TEC from I.P. tumors express PNAd more frequently and at higher levels than TEC from S.C. tumors.

### Lower expression of PNAd on tumor endothelial cells is associated with lower expression of both glycosyl transferases and scaffolding proteins

**Figure 3:**
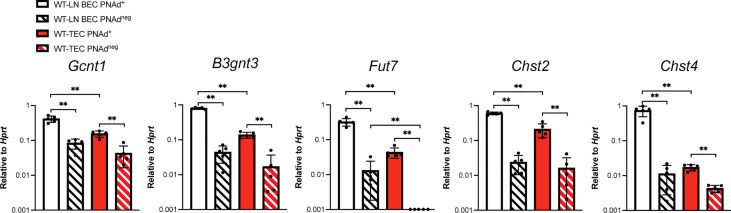
Glycosyl transferases are expressed at significantly lower levels in PNAd^+^ TEC than in LN PNAd^+^ BEC. C57BL/6 mice were I.P. injected with B16-OVA cells and tumors were harvested 14 days after implantation. LN were harvested from non-tumor-bearing mice. Endothelial cells were sorted from CD45+ depleted LN and I.P. tumor suspensions, and the expression levels of indicated RNA transcripts were determined by quantitative PCR (qPCR), as described in Methods. Data from one experiment presented as 2^-ΔCT^ relative to Hprt, n=5 LN or I.P. tumors per group. Results are mean ± SD analyzed by unpaired Welch’s t-test. **p<0.01.

The lower level of PNAd on TEC might reflect lower expression of either glycosyl transferases that synthesize PNAd or scaffolding proteins that display it. We flow sorted PNAd^+^ and PNAd^neg^ cells from I.P. tumors and LN and evaluated expression of these molecules by quantitative PCR (qPCR). Consistent with other work (49, 50), the glycosyl transferases *Gcnt1*, *B3gnt3*, *Fut7*, *Chst2*, and *Chst4* were all expressed at significantly higher levels in PNAd^+^ than PNAd^neg^ LN BEC (**Figure 3**). Similarly, all of these enzymes were expressed at higher levels in PNAd^+^ TEC than in PNAd^neg^ TEC. With the exception of *Fut7*, the expression levels of these glycosyl transferases were not significantly different between PNAd^neg^ LN BEC and PNAd^neg^ TEC (**Figure 3**). However, *Gcnt1*, *B3gnt3*, *Fut7*, and *Chst2* were expressed ~2-7-fold lower in PNAd^+^ TEC than in PNAd^+^ LN BEC (**Figure 3**), consistent with the 6-fold lower gMFI of surface PNAd on PNAd^+^ TEC (**Figure 2**). Most significantly, *Chst4* expression was ~42-fold lower in PNAd^+^ TEC than in PNAd^+^ LN BEC. *Chst4* is selectively expressed in LN high endothelial venules and the major enzyme responsible for the generation of 6-sulfo sialyl Lewis X on the Core 1 *O*-glycan structure of PNAd (39–42).

**Figure 4:**
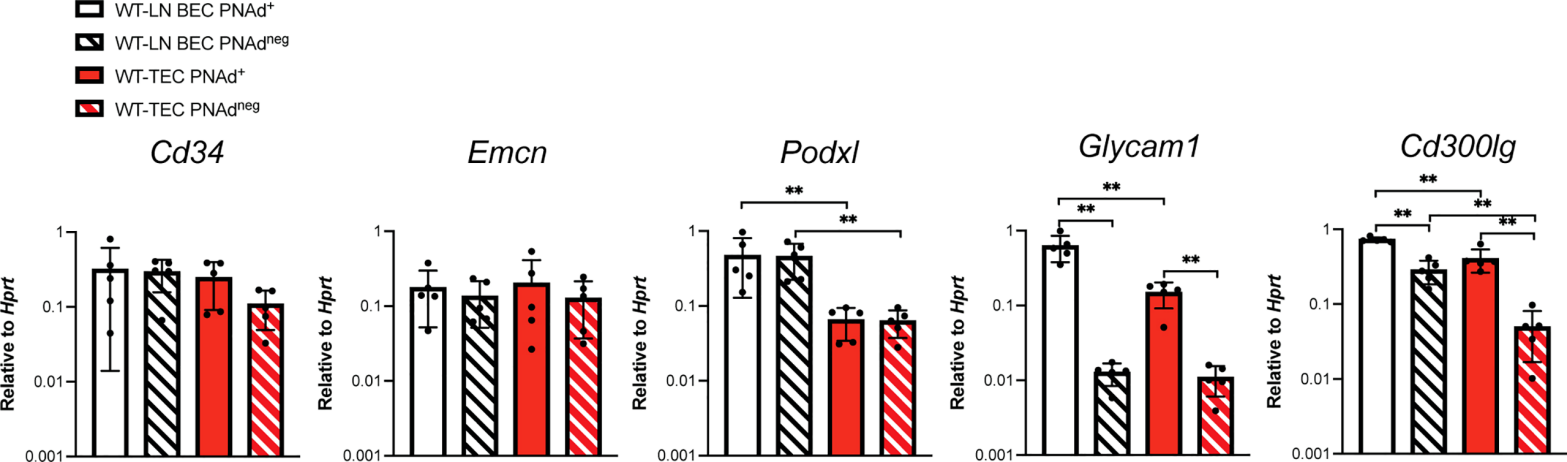
Scaffolding proteins are expressed at significantly lower levels in PNAd^+^ TEC than in LN PNAd^+^ BEC. C57BL/6 mice were I.P. injected with B16-OVA cells and tumors were harvested 14 days after implantation. LN were harvested from non-tumor-bearing mice. Endothelial cells were sorted from CD45+ depleted LN and I.P. tumor suspensions, and the expression levels of indicated RNA transcripts were determined by qPCR, as described in Methods. Data from one experiment presented as 2^-ΔCT^ relative to Hprt, n=5 LN or I.P. tumors per group. Results are mean ± SD analyzed by unpaired Welch’s t-test. **p<0.01, all other paired bar comparisons are not significant (p>0.05) and have been omitted for clarity.

Transcript levels for scaffolding proteins *Cd34* and *Emcn* were comparable between LN BEC and TEC, regardless of whether they expressed PNAd (**Figure 4**). *Podxl* was also expressed comparably by PNAd^+^ and PNAd^neg^ cells from either LN or I.P. tumors, but the transcript levels for this molecule were significantly less in TEC than in LN BEC. On the other hand, *Glycam1* and *Cd300lg* were more highly expressed in PNAd^+^ cells than in PNAd^neg^ cells in both LN BEC and TEC, although as with *Podxl*, they were expressed at significantly lower levels in TEC than in LN BEC (**Figure 4**). The ~2-7-fold differences in expression of *Podxl*, *Glycam1*, and *Cd300lg* between PNAd^+^ LN BEC and PNAd^+^ TEC are again consistent with the 6-fold lower gMFI of surface PNAd on PNAd^+^ TEC. Collectively, these results suggest that PNAd expression on TEC is achieved by upregulation of the same subsets of glycosyl transferases and scaffolding proteins as on LN BEC. However, the magnitude of these upregulations in TEC is significantly lower in every case, and the substantially deficient upregulation of *Chst4* could also affect PNAd structure.

### Tumor necrosis factor receptor signaling regulates the transcript levels of some scaffolding proteins and glycosyl transferases in tumor endothelial cells.

**Figure 5:**
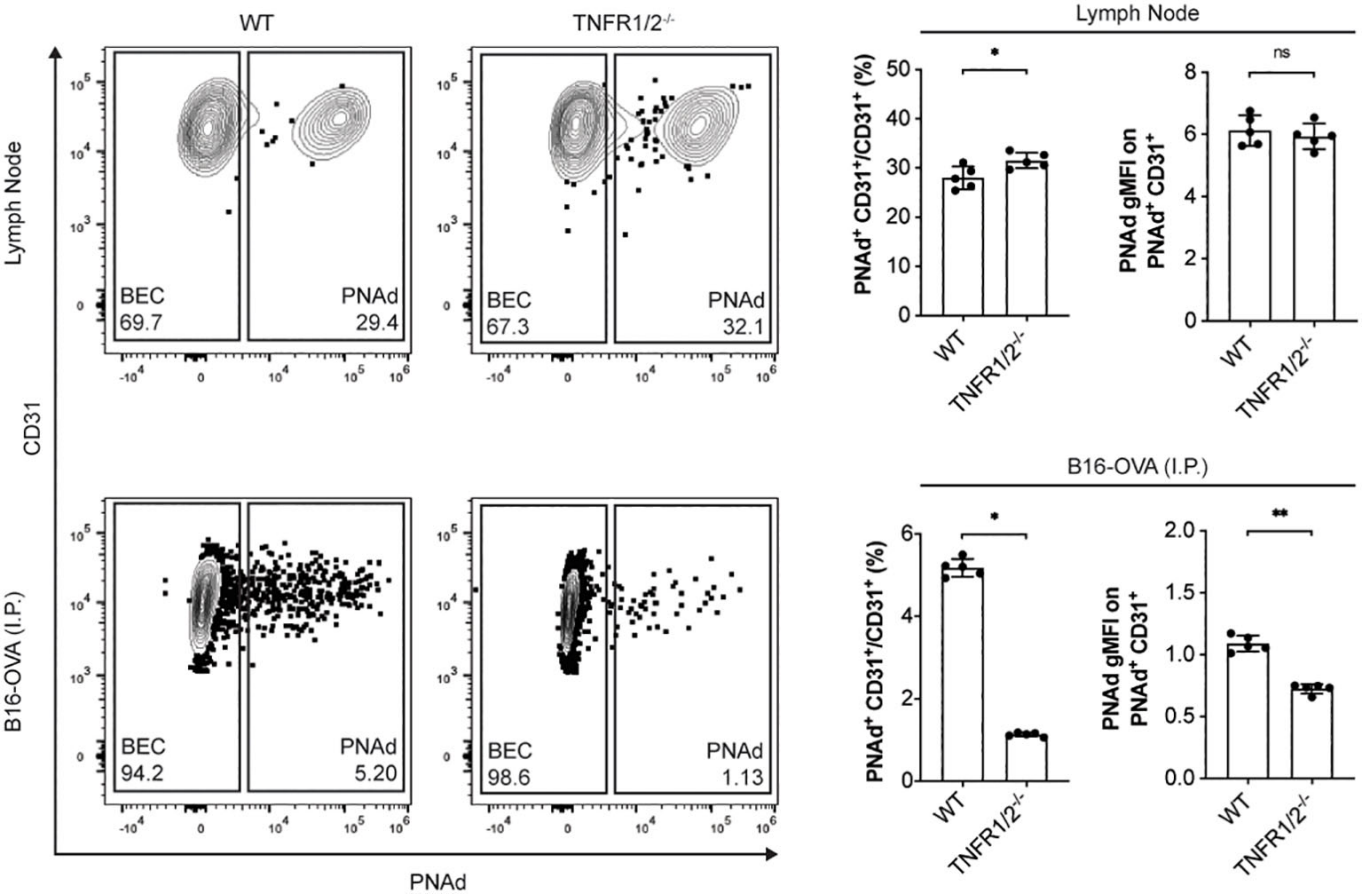
Tumor necrosis factor receptor signaling controls PNAd expression in most TEC but not in LN BEC. C57BL/6 WT or TNFR1/2^-/-^ mice were I.P. injected with B16-OVA cells and tumors were harvested 14 days later. LN were harvested from non-tumor-bearing mice. Resected tissues were prepared for flow cytometry as described in Methods. Representative flow cytometry plot, percentages, and gMFI of PNAd on CD45^neg^ Ter119^neg^ PDPN^neg^ CD31^+^ endothelial cells in CD45^+^ depleted LN and I.P. tumors suspensions. PNAd gMFIs were calculated on cells gated above FMO control. Data represents one experiment, n = 5 LN or tumors per group. Results are mean ± SD analyzed by unpaired Welch’s t-test. ns: p>0.05, *p<0.05, **p<0.01.

**Figure 6:**
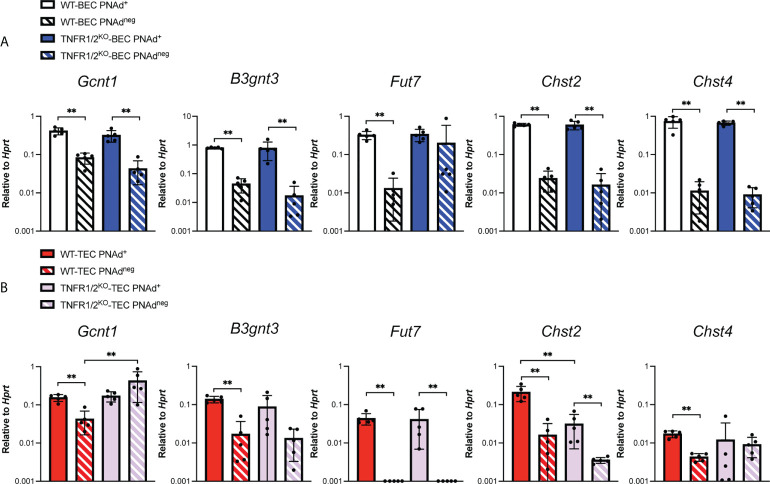
Tumor necrosis factor receptor signaling regulates *Chst2* expression in PNAd^+^ TEC. C57BL/6 WT or TNFR1/2^-/-^ mice were I.P. injected with B16-OVA cells and tumors were harvested 14 days after implantation. LN were harvested from non-tumor-bearing mice. **(A, B)** Endothelial cells were sorted from CD45^+^ depleted LN and I.P. tumor suspensions, and the expression levels of indicated RNA transcripts were determined by qPCR, as described in Methods. Data from one experiment presented as 2^-ΔCT^ relative to Hprt, n=5 LN or I.P. tumors per group. **(A, B)** Results are mean ± SD analyzed by unpaired Welch’s t-test. **p<0.01, all other paired bar comparisons are not significant (p>0.05) and have been omitted for clarity.

**Figure 7:**
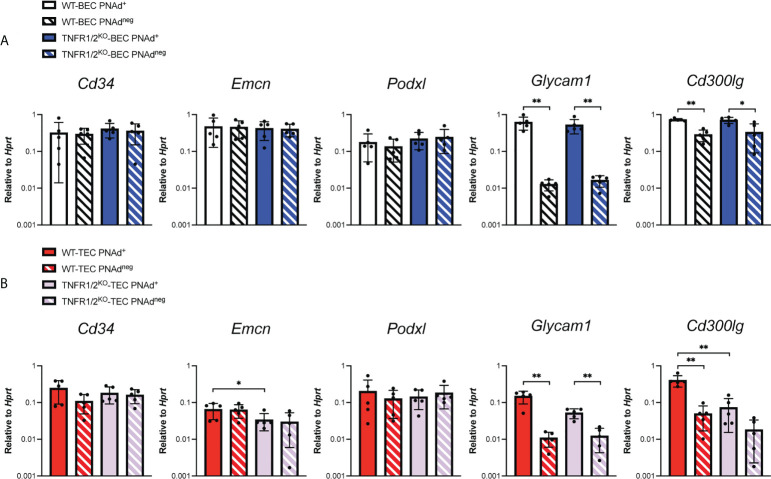
Tumor necrosis factor receptor signaling regulates *Podxl* and *Cd300lg* expression in PNAd^+^ TEC. C57BL/6 WT or TNFR1/2^-/-^ mice were I.P. injected with B16-OVA cells and tumors were harvested 14 days after implantation. LN were harvested from non-tumor-bearing mice. **(A, B)** Endothelial cells were sorted from CD45^+^ depleted LN and I.P. tumor suspensions, and the expression levels of indicated RNA transcripts were determined by qPCR, as described in Methods. Data from one experiment presented as 2^-ΔCT^ relative to Hprt, n=5 LN or I.P. tumors per group. (A-B) Results are mean ± SD analyzed by unpaired Welch’s t-test. *p<0.05, **p<0.01, all other paired bar comparisons are not significant (p>0.05) and have been omitted for clarity.

PNAd expression on LN BEC depends on signaling through LTβR (31, 51, 52) but not TNFR (**Figure 5**). In contrast, PNAd is not detected by IF on the vasculature of I.P. tumors grown in TNFR1/2^-/-^ mice, and its expression does not depend on LTβR signaling (31). By flow cytometry, only a small percentage of TEC from I.P. tumors grown in TNFR1/2^-/-^ mice retained PNAd, although their surface expression level was lower than that of TEC from wild-type (WT) mice (**Figure 5**). This confirms that PNAd expression in most TEC depends on TNFR signaling, while a small subset of TEC apparently depends on an alternative signaling pathway. Using these cells, we determined how the loss of TNFR signaling altered expression of genes involved in PNAd biosynthesis. As expected from **Figure 5**, the expression of genes encoding glycosyl transferases (**Figure 6**) and scaffolding proteins (**Figure 7**) in PNAd^+^ and PNAd^neg^ LN BEC was unaltered in TNFR1/2^-/-^ mice relative to their WT counterparts. In PNAd^neg^ TEC from I.P. tumors grown in TNFR1/2^-/-^ mice, there was no reduction in the expression of genes encoding scaffolding proteins (**Figure 7**) or most glycosyl transferases, although the expression of *Gcnt1* increased (**Figure 6**). This suggests that the TNFR-induced expression of PNAd on TEC in WT mice is a consequence of upregulation of the genes identified in **Figure 3** and **4**. In the small subset of PNAd^+^ TEC from I.P. tumors grown in TNFR1/2^-/-^ mice, *Chst2* expression was 7-fold lower, but no other biosynthetic enzyme, including *Chst4*, was significantly changed (**Figure 6**). Also, *Podxl* and *Cd300lg* expression was lower by 2- and 6-fold, respectively, and there was a trending but insignificant reduction in *Glycam1* (**Figure 7**). These results are consistent with the possibility that an alternative signaling pathway, operating in the absence of TNFR signaling, upregulates most of the relevant genes identified in **Figures 3** and **4**, but is selectively deficient in its ability to upregulate *Chst2*, *Podxl*, and *Cd300lg*, and potentially *Glycam1*.

### Checkpoint immunotherapy augments PNAd expression on I.P. tumor vasculature

**Figure 8:**
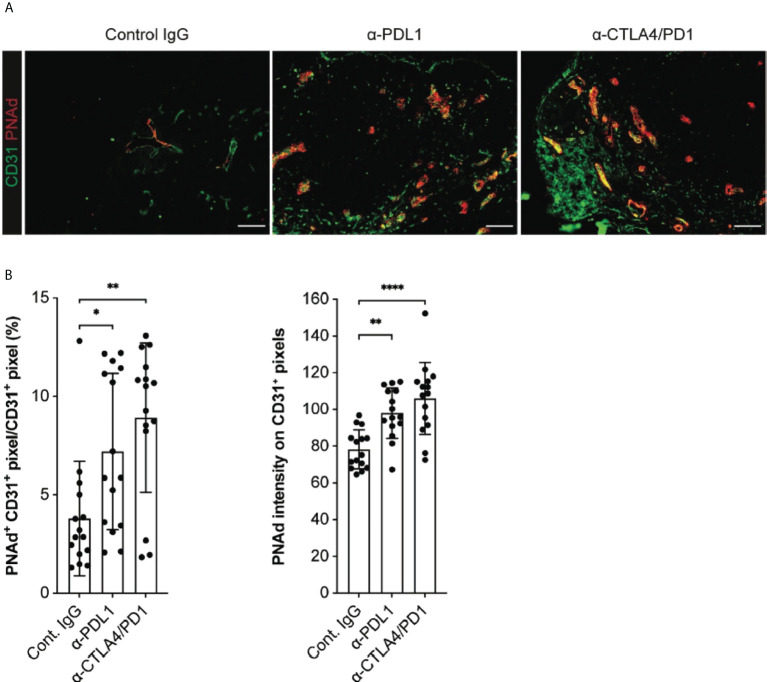
Checkpoint immunotherapy augments PNAd expression on I.P. tumor vasculature. C57BL/6 mice were I.P. injected with B16-OVA cells and tumor-bearing mice were treated with control IgG, anti-PDL1, or anti-CTLA4/PD1 beginning 3 days after implantation. Tumors were harvested 14 days after implantation and prepared for IF as described in Methods. **(A)** Representative images of tyramide signal amplified tumor sections stained with indicated markers. Scale bar = 100 µm. **(B)** Quantitative image summary data for amplified tissue sections. PNAd percentages and pixel intensities were calculated on a CD31^+^ mask. Data represents three experiments, n = 15 individual sections per group. **(B)** Results are mean ± SD analyzed by unpaired Welch’s t-test. *p<0.05, **p<0.01, and ****p<0.0001, all other paired bar comparisons are not significant (p>0.05) and have been omitted for clarity.

Checkpoint immunotherapy has also been associated with enhanced expression of HR ligands on the tumor vasculature, such as ICAM-1 and VCAM-1 (55, 56). To determine whether checkpoint immunotherapy altered the expression of PNAd on tumor vasculature, we treated I.P. tumor-bearing WT mice with either anti-PD-L1 monotherapy or the combination of anti-CTLA4 and anti-PD1 and analyzed PNAd expression after 14 days of outgrowth. Both treatments significantly increased the percentage of CD31^+^ TEC pixels that co-stained with PNAd (**Figures 8A, B** **)**. Also, the staining intensity of PNAd on CD31^+^ TEC pixels was significantly higher in treated tumors. Despite these increases, PNAd staining remained localized with tumor associated tertiary lymphoid structures (TA-TLS), and was not found on non-TA-TLS associated vasculature (57). Previously, we demonstrated that checkpoint immunotherapy augmented the number of T-cells in I.P. tumors (57). Since PNAd expression in I.P. tumors depends on effector CD8 T cells secreting LTα_3_ (31), these results suggest that checkpoint immunotherapy augments PNAd expression on the tumor vasculature by increasing the representation of CD8 T cells secreting LTα_3_ in tumors, which in turn enhances the expression of PNAd associated components.

## Discussion

In this report, we identified differences in the expression of genes encoding scaffolding proteins and glycosyl transferases involved in biosynthesis of PNAd that could account for its lower expression level in TEC, and its dependence on TNFR signaling. We found that TEC and LN BEC upregulated the same subsets of molecules in association with PNAd expression, but the extent of upregulation was less in PNAd^+^ TEC than in LN PNAd^+^ BEC, consistent with the lower level expression of PNAd on tumor endothelium. The glycosyl sulfotransferase *Chst4* was particularly poorly induced, likely contributing to a significant underrepresentation of mature 6-sulfo sialyl Lewis X glycans on both Core 1 and Core 2 branches. Previously, we demonstrated that PNAd expression on the tumor vasculature was controlled by a mechanism involving intratumoral effector CD8 T cells secreting LTα_3_, which signaled through TNFR on TEC (31). Here, we showed that loss of TNFR signaling led to PNAd^neg^ cells that were indistinguishable from their WT counterparts, suggesting that TNFR signaling regulates the same PNAd associated genes as LTβR signaling in LN BEC. However, we also found a remnant population of PNAd^+^ TEC with normally upregulated expression of most of these genes, but impaired upregulation of genes encoding the scaffolding proteins *Podxl* and *Cd300lg* and the glycosyl sulfotransferase *Chst2*, suggesting the operation of a 3^rd^ pathway for PNAd induction. Finally, we showed that the percentage of PNAd^+^ TEC and their surface expression level were increased by checkpoint immunotherapy treatment. This work provides insight into the mechanisms regulating PNAd biosynthesis in TEC and provides a platform to enhance its expression to support a continual influx of naïve cells, sustaining anti-tumor immunity.

Our results provide new insights into the control of the biosynthetic pathway leading to expression of 6-sulfo-sialyl Lewis X on TEC. We found that *B3gnt3*, *Gcnt1*, *Fut7*, *Chst2*, and *Chst4* were expressed at higher levels in PNAd^+^ TEC relative to their PNAd^neg^ counterparts. This is consistent with their elevated expression in PNAd^+^ vs. PNAd^neg^ LN BEC (49, 50), and their essential roles in PNAd biosynthesis in these cells (33). However, while *Chst2* and *Chst4* were comparably expressed in PNAd^+^ LN BEC, *Chst4* was significantly under expressed relative to *Chst2* in PNAd^+^ TEC. Previous studies using knockout mice have established that *Chst2* and *Chst4* play complementary and partially redundant roles in PNAd expression in LN (39, 40). *Chst4* is primarily responsible for PNAd that is expressed on the luminal endothelial surface (39), while *Chst2* contributes to PNAd expressed on both luminal and abluminal surfaces (40–42). Our previous observation of PNAd expression on both surfaces of tumor endothelium (31) is consistent with the very low level of *Chst4* expression in TEC, and suggests that *Chst2* is largely responsible for synthesizing 6-sulfo-sialyl Lewis X in TEC. The MECA-79 antibody detects 6-sulfo-sialyl Lewis X in the Core 1, but not Core 2, *O*-glycan biantennary branch (37). Since *Chst2* mediates GlcNAc sulfation on the Core 1 branch inefficiently (41), the levels of 6-sulfo-sialyl Lewis X on TEC may be higher than is suggested by MECA-79. PNAd structures on TEC may also be largely “single-armed”, with only Core 2 derivatized by 6-sulfo-sialyl Lewis X. Regardless, PNAd expression on Peyer’s Patch endothelium is entirely dependent on *Chst2* (39, 40). This, together with our earlier work (31, 32), demonstrates that these structures support naïve lymphocyte entry.

We found that PNAd^+^ TEC and LN BEC also express similar scaffolding proteins that have been shown to display PNAd and regulate them similarly. *Cd34* and *Emcn* were expressed comparably by PNAd^+^ and PNAd^neg^ endothelial cells from both LN and I.P. tumors. In contrast, *Podxl* expression also did not differ between PNAd^+^ and PNAd^neg^ endothelial cells in either tissue, but its overall level of expression was substantially higher in LN BEC than TEC. Finally, *Glycam1* and *Cd300lg* were expressed at higher levels in PNAd^+^ than in PNAd^neg^ cells in both tissues, and again, the transcript levels for these molecules were significantly less in PNAd^+^ TEC than in PNAd^+^ LN BEC. These scaffolding proteins are redundant with one another in PNAd display, and at least partially redundant in promoting L-selectin engagement with PNAd, as mice deficient in *Cd34* (58) or *Glycam1* (33) show no impaired trafficking of naïve T cells to peripheral LN. However, together with the lower expression levels of multiple glycosyl transferases, particularly *Chst4*, the overall lower level of *Podxl*, *Glycam1*, and *Cd300lg* in PNAd^+^ TEC may also contribute to the lower level of PNAd expression on the tumor vasculature.

This report also identified components of the PNAd biosynthesis pathway in TEC that are regulated by TNFR signaling. The number of PNAd^+^ TEC is reduced by about 80% in TNFR1/2^-/-^ mice. The resulting PNAd^neg^ cells showed no reduction in any evaluated glycosyl transferases or scaffolding proteins and are thus indistinguishable from their counterparts in tumors from WT mice. By comparison of gene expression in PNAd^+^ and PNAd^neg^ LN BEC and TEC, this suggests that TNFR signaling induces PNAd by acting on the same genes as LTβR signaling does in LN BEC, albeit less efficiently, and with a particular deficiency in upregulation of *Chst4.* However, a small percentage of TEC from these tumors continue to express a lower level of PNAd. These cells selectively express lower levels of *Chst2*, *Podxl*, and *Cd300lg*, and potentially *Glycam1*, all of which are also elevated in PNAd^+^ cells vs PNAd^neg^ cells from WT mouse tumors. It is possible that TEC that retain PNAd in the absence of TNFR signaling are responsive to signals from LTβR as an alternative. However, in PNAd^+^ LN BEC, LTβR signaling upregulates *Chst2* and *Glycam1*, but also *Gcnt1*, *Chst4*, *Fut7*, and not *Podxl* or *Cd300lg* (51, 52). This difference in response pattern seems inconsistent with the hypothesis that the remnant fraction of PNAd^+^ TEC in TNFR1/2^-/-^ mice are regulated by LTβR signaling. Nonetheless, these results demonstrate a distinct regulation of PNAd biosynthetic components by TNFR1/2 and LTβR signaling. the mutually exclusive responses of LN BEC and TEC to these two different signals also point to important anatomic microenvironmental controls on endothelial cell signaling responsiveness.

Related to this, the reasons that all evaluated PNAd glycosyl transferases and several scaffolding proteins are expressed at significantly lower levels in PNAd^+^ TEC than in LN PNAd^+^ BEC are unclear. It is possible that this reflects the relative efficiency with which the TNFR and LTβR signaling pathways engage transcription factors that upregulate these genes. On the other hand, several studies have also shown that the epigenetic profile of BEC is different from TEC (reviewed in (59). Two studies demonstrated that human umbilical vein endothelial cells cultured with conditioned tumor media underwent epigenetic modifications that resulted in reduced VCAM-1 and ICAM-1 expression (60, 61). Thus, epigenetic modifications that occur in the tumor microenvironment could also reduce upregulation of PNAd associated components in TEC. Finally, it is possible that this is controlled by the immunosuppressive microenvironment of the tumor. In murine methylcholanthrene-induced fibrosarcoma, regulatory T cells have been shown to limit PNAd expression on tumor vessels (62, 63), although the mechanism of action is unknown. Conversely, checkpoint immunotherapy has been associated with enhanced expression of HR ligands, such as ICAM-1 and VCAM-1, on tumor vasculature (55, 56), and we found that anti-PD-L1 monotherapy or the combination of anti-CTLA4 and anti-PD1 enhanced the fraction of CD31^+^ TEC expressing PNAd and their surface levels of PNAd. However, the fraction of PNAd^+^ CD31^+^ TEC and their PNAd expression level in treated tumors still did not reach that of LN BEC. In related work, we demonstrated that these therapies also led to an increase in the number and size of TA-TLS, but we found no evidence for PNAd expression on non-TA-TLS associated tumor vasculature (57). Since PNAd expression in I.P. tumors depends on effector CD8 T cells secreting LTα_3_ (31), and these therapies also enhanced the representation of T-cells in I.P. tumors (57), this suggests that immunotherapy-augmented PNAd expression on the tumor vasculature is driven by an increased number of intratumoral CD8 T cell effectors secreting LTα_3_. It also suggests that the increased fraction of TEC expressing PNAd create nucleation sites for the formation of new TA-TLS, or the expansion of existing ones. Determining the factors that limit the expression of PNAd scaffolding proteins and glycosyl transferases could identify targets for enhancing the expression of these molecules to enhance naïve and central memory cell infiltration into tumors, promote the development and/or expansion of tertiary lymphoid structures, and augment anti-tumor immunity.

## Materials and methods

### Mice

Female C57BL/6 mice were from the National Cancer Institute. TNFR1/2^-/-^ mice were from the Jackson Laboratory. All mice were bred and maintained in specific pathogen-free conditions. All experiments were carried out on female mice that were ~8-12 weeks of age. All protocols and experiments were approved by the University of Virginia Institutional Animal Care and Use Committee.

### Tumor cell lines

B16-OVA mouse melanoma cells expressing recombinant ovalbumin has previously been described (64). B16-OVA tumor cells were cultured at 37°C and 5% CO_2_ in RPMI-1640 (Corning) containing 10% (v/v) fetal bovine serum, 2 mM L-glutamine (ThermoFisher Scientific), and 15 mM HEPES (ThermoFisher Scientific).

### Tumor induction and treatment mice

Tumor cells (4 x 10^5^) were I.P. or S.C. (loose neck scruff) injected into mice and allowed to establish for 14 days prior to harvest. For checkpoint immunotherapy experiments, either monotherapy anti-PDL1 (250 µg per injection, 10F.9G2, BioXcell) or dual therapy anti-PD1 (250 µg per injection, RMP1-14, BioXcell) and anti-CTLA4 (250 µg per injection, 9D9, BioXcell) was injected I.P. into tumor-bearing mice three days after tumor implantation and then every three days until tumor harvest.

### Digestion of resected tissues

Resected LN and tumors were minced and digested with a solution of 0.1 mg/ml DNase I (Sigma), 0.8 mg/ml Collagenase Dispase (Sigma), and 0.2 mg/ml Collagenase P (Sigma) for 30 minutes at 37°C. Every 5 minutes, tissue suspensions were pipetted up-and-down several times. Digested tissues were depleted of red blood cells using RBC Lysing Buffer Hybri-Max (Sigma) according to manufacturer’s instructions.

### Enrichment of cells from digested tissues

Digested tumor suspensions were depleted of hematopoietic cells using CD45 magnetic beads (Miltenyi Biotec) on an AutoMACS Pro Separator (Miltenyi Biotec) according to manufacturer’s instructions. CD45^+^ depleted suspensions were stained with biotinylated anti-CD31 (0.5 µg/mL) for 15 minutes at 4°C and enriched using anti-biotin magnetic beads (Miltenyi Biotec) on an AutoMACS Pro Separator according to manufacturer’s instructions. BEC and TEC were sorted to highest purity according to the procedure below.

### Flow cytometry and cell sorting

Cell surface staining was done in PBS containing 2% FBS, 2 mM EDTA (Sigma), and 2 mM NaN_3_ (Sigma) for 30 minutes at 4°C. Live/Dead Aqua (Invitrogen) or 4,6-diamidino-2-phenylindole (Sigma) were used to exclude dead cells from analysis. Endothelial cells were defined as live, singlet, Ter119^neg^, CD45^neg^, PDPN^neg^, CD31^+^. Samples were run on a FACSCanto II (BD) or Attune NxT (ThermoFisher/Invitrogen) and analyzed using FlowJo Software (BD Bioscience). For qPCR experiments, pre-enriched endothelial cell populations were sorted on an Influx Cell Sorter (BD) directly into RNAlater Stabilization Solution (ThermoFischer Scientific) or PBS. A small aliquot of the sorted population was re-run to determine ~95% purity.

### Antibodies for flow cytometry

**Table:** 

Antibody	Vendor	Clone	Catalog #	RRID
APC-Cy7 anti-mouse CD45	Biolegend	30-F11	102515	AB_2161030
PerCP-eFlour 710 anti-mouse CD31	ThermoFisher	390	46-0311-82	AB_1834429
Biotin anti-mouse CD31	Biolegend	390	102404	AB_312899
PE-Cy7 anti-mouse podoplanin (gp38)	Biolegend	8.1.1	127412	AB_10613648
Biotin anti-mouse podoplanin (gp38)	Biolegend	8.1.1	127404	AB_1134222
Alexa Fluor 488 PNAd	ThermoFischer	MECA-79	**53-6036-82**	AB_10804391

### Four-color IF microscopy

Preparation of tumor tissue for IF microscopy using formalin fixation and cutting frozen blocks on a -20°C cryostat has previously been described (65). Tumor sections were incubated in 100% methanol for 10 minutes at -20°C. Then, slides were immersed in PBS for 10 minutes at room temperature. For Fc blocking, tumor sections were incubated with 0.5 μg/mL anti-CD16/32 (BioXcell; Clone:2.4G2) unconjugated antibody in PBS containing 5% BSA (Sigma) and 0.3% Triton X-100 (Sigma) for 15 minutes at room temperature. Endogenous biotin in tumor sections was blocked using the Avidin/Biotin Blocking kit (Vector Laboratories) according to manufacturer’s instructions. Endogenous peroxidases in tumor sections were quenched with PBS containing 3% hydrogen peroxide and 0.1% (w/v) sodium azide for 45 minutes at room temperature. Tumor sections were incubated overnight at 4°C with primary antibodies in either PBS containing 3% hydrogen peroxide and 0.1% (w/v) sodium azide or TNB Blocking Buffer (PerkinElmer) if performing tyramide signal amplification. The TSA Biotin Kit (Perkin Elmer) was used according to manufactures instructions to amplify biotinylated labelled tumor sections. Tumor sections are counterstained with fluorescently conjugated secondary antibodies and/or streptavidin in PBS containing 3% hydrogen peroxide and 0.1% (w/v) sodium azide for 2 hours at room temperature. Prior to imaging, tumor sections are mounted with ProLong Gold Antifade Mountant with or without DAPI (ThermoFisher Scientific).

### Reagents for four-color IF microscopy

**Table:** 

Antibody	Vendor	Clone	Catalog #	RRID
Alexa Fluor 647 anti-mouse CD31	Biolegend	MEC13.3	102516	AB_2161029
Biotin anti-mouse/human PNAd	Biolegend	MECA-79	120804	AB_493557
DyLight™ 550 Streptavidin	ThermoFisher	N/A	**84542**	–

### Four-color IF image acquisition

One section per tumor was evaluated and all images were captured on an AxioImager with Apotome (Zeiss). Fluorescence minus one (FMO) or isotype staining controls were used to establish thresholds and exposure times to visualize positive signals while minimizing background fluorescence. Fifteen-20 low magnification images per tumor section were captured and stitched together using ImageJ Software (NIH) to create an image of the entire tumor section. Values across respective regional images were averaged together for each tumor section. In all experiments, the quantification of CD31^+^ and PNAd^+^ areas were performed using ImageJ software (NIH) on original fluorescence images taken at identical exposures. Consistent thresholds were applied to each image to identify CD31^+^ and PNAd^+^ pixels. The percentages and gMFI of PNAd^+^ pixels within the region of interest of CD31^+^ area was calculated using ImageJ Software (NIH). For image presentation, brightness and contrast were linearly adjusted and color-merged images were generated using Photoshop CS6 Software (Adobe).

### Quantitative RT-PCR

RNA was purified from flow sorted endothelial cells using RNEasy kits (Qiagen). High-Capacity cDNA Reverse Transcription Kit (Applied Biosystems) and purified RNA was used to generate cDNA. Amplification was performed using TaqMan Fast Advanced Master Mix (Applied Biosystems) and QuantStudio 6 Flex Real-Time PCR system (Applied Biosystems) with the following program: 50°C for 2 minutes; 95°C for 2 minutes; 40 cycles of 95°C for 1 second, 60°C for 20 seconds.

### Probes for quantitative RT-PCR

**Table:** 

Gene	Vendor	Assay ID
*Podxl*	ThermoFischer	Mm00628472_m1
*Cd34*	ThermoFischer	Mm00519283_m1
*Emcn*	ThermoFischer	Mm00497495_m1
*Glycam1*	ThermoFischer	Mm00801716_m1
Cd300lg	ThermoFischer	Mm01266006_m1
*Chst2*	ThermoFischer	Mm00490018_g1
*Chst4*	ThermoFischer	Mm00488783_s1
*Gcnt1*	ThermoFischer	Mm02010556_s1
*B3gnt3*	ThermoFischer	Mm00472247_g1
*Fut7*	ThermoFischer	Mm04242850_m1

### Statistical analyses

Statistical details of each experiment in this work are reported in the figure legends. Normality of data distribution was determined by D’Agostino-Pearson omnibus normality test and variance between groups was assessed by the *F*-test. P-values for the comparison between two independent groups were calculated by Welch’s t-test. Error bars shown in graphical data represents mean ± standard deviation (S.D.) for normally distributed data. P<0.05 was considered statistically significant. All statistics were calculated using Graph Pad Prism version 7.0, R version 4, and the SAS software suite version 9.4.

## Data availability statement

The original contributions presented in the study are included in the article/supplementary material. Further inquiries can be directed to the corresponding author.

## Ethics statement

The animal study was reviewed and approved by University of Virginia Institutional Animal Care and Use Committee.

## Author contributions

Experiments involving tumor implantation, flow cytometry, endothelial cell sorting, and quantitative PCR were performed and analyzed by AR and GP. Immunofluorescence image capture and analysis were performed by AR. VE supervised all experiments associated with this project and participated in experimental design and interpretation of results. All authors contributed to the article and approved the submitted version.

## Funding

This work was supported by the United States Public Health Service (USPHS) Grants CA78400 and CA181794, the University of Virginia (UVA) Cancer Center Schiff Foundation Grant, USPHS Immunology Training Grant AI007496, USPHS Cancer Center Support Grant P30 CA44579, and a Robert Wagner Fellowship from the University of Virginia School of Medicine.

## Acknowledgments

We thank all the members of Dr. Victor H. Engelhard and Dr. Andrew Dudley laboratories for insightful discussion and suggestions. We also thank Dr. Kenneth Tung and members of his laboratory for aid in developing immunofluorescence protocols for capturing PNAd expression on tumor endothelial cells. Lastly, we thank the University of Virginia School of Medicine Histology Core for tissue sectioning and the Flow Cytometry Core for cell sorting experiments.

## Conflict of interest

The authors declare that the research was conducted in the absence of any commercial or financial relationships that could be construed as a potential conflict of interest.

## Publisher’s note

All claims expressed in this article are solely those of the authors and do not necessarily represent those of their affiliated organizations, or those of the publisher, the editors and the reviewers. Any product that may be evaluated in this article, or claim that may be made by its manufacturer, is not guaranteed or endorsed by the publisher.
